# IgG4 Kinetics in Ultra‐Rush Vespula Venom Immunotherapy: How Early Is the Response?

**DOI:** 10.1111/all.16648

**Published:** 2025-07-17

**Authors:** Matteo Martini, Barbara Cinti, Ilaria Claudi, Maria Chiara Braschi, Yara Aboud, Elena Buti, Davide Palmeri, Maria Giovanna Danieli, Maria Beatrice Bilò

**Affiliations:** ^1^ Allergy Unit, Department of Internal Medicine University Hospital AOU Delle Marche Ancona Italy; ^2^ Department of Clinical and Molecular Sciences Marche Polytechnic University Ancona Italy; ^3^ Laboratory Medicine University Hospital AOU Delle Marche Ancona Italy; ^4^ Post‐Graduate School of Allergy and Clinical Immunology Marche Polytechnic University Ancona Italy; ^5^ Marche Polytechnic University Ancona Italy; ^6^ Department of Internal Medicine, Immunology of Rare Diseases and Transplants Unit University Hospital AOU Delle Marche Ancona Italy

**Keywords:** allergen‐specific immunoglobulins, hymenoptera venom allergy, IgG4, immune tolerance, kinetics, systemic reactions, ultra‐rush protocol, venom immunotherapy

To the Editor,

Hymenoptera venom allergy (HVA) can trigger severe systemic reactions [1].

Venom immunotherapy (VIT) is the only treatment that reduces the future risk of anaphylaxis [2]. Although no validated biomarkers are available to predict its effectiveness, the increase of serum immunoglobulin subclass 4 (IgG4) levels during VIT was suggested to be a protective surrogate biomarker [3]. However, limited data are available on the kinetics of IgG4.

This was a prospective, longitudinal study to investigate the kinetics of IgG4 to Vespula venom and to its molecular allergens (Ves v 1 and Ves v 5) at different timepoints after VIT was administered with a 2‐day ultra‐rush protocol (Table S1).

Twenty‐four patients with a confirmed allergy to Vespula venom and eligible for VIT were consecutively enrolled at the Allergy Unit of University Hospital AOU delle Marche (Italy).

Their baseline characteristics are reported in Table S2. IgG4 levels were measured at five time points: pre‐VIT, end of the second day of ultra‐rush, 1 week, 1 month, and 6 months post‐VIT initiation. The Kruskal–Wallis test was performed (alpha = 0.05), with STATA v.18 (StataCorp LLC, Texas, USA), to compare IgG4 levels. Since all the interventions were part of routine clinical practice, a formal approval by the Ethics Committee was not needed, and all the patients signed the informed consent for the standard clinical procedures.

The results showed a significant increase of IgG4 to Vespula venom, approximately 2‐fold, 6‐fold, and 8‐fold at 1 week, 1 month, and 6 months post‐VIT, respectively (*p* < 0.001) (Figure 1). Conversely, no changes were observed after 24 h. The increase of IgG4 was statistically significant also for Ves v 1 (*p* = 0.014) and Ves v5 (*p* = 0.002), with similar kinetics and no significant differences between Ves v1, Ves v5, or the whole extract. No significant correlations were observed between IgG4 kinetics and patients' characteristics.

**FIGURE 1:**
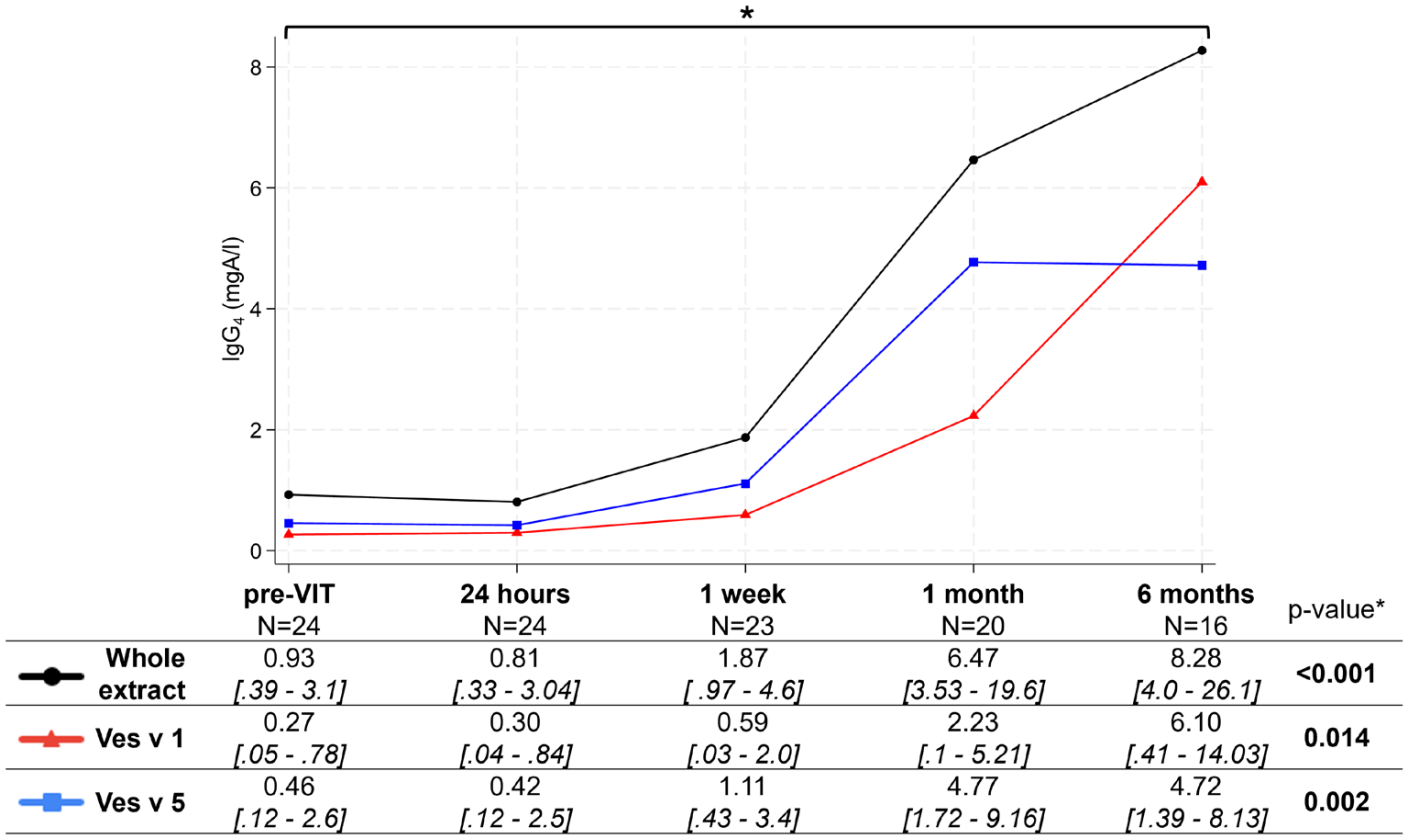
Median IgG4 levels before and after 2‐day ultra‐rush vespula venom immunotherapy (*N* = 24). Data are medians [interquartile range]. mgA/L, milligrams of antigen‐specific antibodies/liter; N, number of patients; VIT, venom immunotherapy. *Kruskal–Wallis test.

These findings demonstrated that ultra‐rush VIT induces a rapid and sustained IgG4 increase, supporting the hypothesis of a very quick onset of the VIT‐induced protection. Furthermore, the similar kinetics of the analyzed allergens suggest a generalized immune response, rather than antigen‐specific variations.

Our results are consistent with previous studies demonstrating early and sustained IgG4 increase after VIT, with variability depending on VIT protocols and the venom type [4].

However, this is the first study investigating the IgG4 kinetics at early timepoints (i.e., end of the second day of ultra‐rush and 1 week after VIT) and with a 2‐day ultra‐rush protocol. Unfortunately, ethical constraints prevent sting challenge validation in Italy, limiting the direct assessment of the early protective effects of VIT. Nonetheless, sting challenges performed in other studies have confirmed the early effectiveness of VIT after reaching the maintenance dose [5] [6]. In addition, this early efficacy is suggested by in‐field stings in other patients who were previously treated with the same venom and protocol in our clinical practice.

In conclusion, the 2‐day ultra‐rush protocol resulted in a significant increase of IgG4 levels to the whole vespula extract, Ves v 1, and Ves v 5. The increase starts already 1 week after reaching the VIT maintenance dose and continues for up to 6 months. These findings may support the early efficacy of VIT, even shortly after initiation. However, further studies on larger patient cohorts and sting challenges would be necessary to confirm these findings.

## Author Contributions

Conceptualization, M.B.B.; formal analysis, M.M.; data management, Y.A., E.B., M.M.; laboratory testing, B.C.; clinical visits, M.B.B., M.C.B., D.P., I.C., E.B., M.M.; writing original draft, M.B.B., M.M., I.C.; all authors reviewed and approved the manuscript.

## Conflicts of Interest

The authors declare no conflicts of interest.

## Supporting information


Data S1

## Data Availability

The data that supports the findings of this study are available in the Supporting Information of this article.

## References

[1] M. B. Bilò , M. G. Danieli , G. Moroncini , and M. Martini , “Hymenoptera Venom Allergy and Anaphylaxis,” Current Pharmaceutical Design 29, no. 3 (2023): 165–177. 35980057 10.2174/1381612828666220817091039

[2] G. J. Sturm , E.‐M. Varga , G. Roberts , et al., “EAACI Guidelines on Allergen Immunotherapy: Hymenoptera Venom Allergy,” Allergy 73, no. 4 (2018): 744–764. 28748641 10.1111/all.13262

[3] D. B. K. Golden , I. D. Lawrence , R. H. Hamilton , A. Kagey‐Sobotka , M. D. Valentine , and L. M. Lichtenstein , “Clinical Correlation of the Venom‐Specific IgG Antibody Level During Maintenance Venom Immunotherapy,” Journal of Allergy and Clinical Immunology 90, no. 3 (1992): 386–393. 1527321 10.1016/s0091-6749(05)80019-3

[4] J. Matysiak , E. Matuszewska , M. L. Kowalski , S. W. Kosiński , E. Smorawska‐Sabanty , and J. Matysiak , “Association Between Venom Immunotherapy and Changes in Serum Protein—Peptide Patterns,” Vaccine 9, no. 3 (2021): 249. 10.3390/vaccines9030249 PMC8001044 33809001

[5] C. Schrautzer , L. Arzt‐Gradwohl , D. Bokanovic , et al., “A Safe and Efficient 7‐Week Immunotherapy Protocol With Aluminum Hydroxide Adsorbed Vespid Venom,” Allergy 75, no. 3 (2020): 678–680. 31394011 10.1111/all.14012 PMC7078896

[6] A. Goldberg and R. Confino‐Cohen , “Bee Venom Immunotherapy – How Early Is It Effective?,” Allergy 65, no. 3 (2010): 391–395. 19839973 10.1111/j.1398-9995.2009.02198.x

