# Supplementary material for: IgG4 Kinetics in Ultra‐Rush Vespula Venom Immunotherapy: How Early Is the Response?
Source: Allergy. 2025 Jul 17;80(10):2902–3. doi: 10.1111/all.16648 (PMC12486365; doi:10.1111/all.16648)
Supplement: Supplementary file 1 — Data S1 [file ALL-80-2902-s001.docx]

**Supplementary materials**

**Table S1- Ultra-rush protocol used to achieve the 100 μg total maintenance dose of venom specific immunotherapy: induction phase.**

| **Day** | **Concentration** | **Administered volume** | **Amount of venom** |
| --- | --- | --- | --- |
| Day 1 | 1 μg/ml | 0,1ml | 0,1 μg |
|  | 10 μg /ml | 0,1ml | 1 μg |
|  | 100 μg /ml | 0,1ml | 10 μg |
|  | 100 μg /ml | 0,2ml | 20 μg |
|  |  |  | **Totale: 31,1 μg** |
| Day 2 | 100 μg/ml | 0,35ml | 35 μg |
|  | 100 μg/ml | 0,35ml | 35 μg |
|  | 100 μg/ml | 0,30ml | 30 μg |
|  |  |  | **Total: 100 μg** |

*This two-day ultra-rush induction phase  was followed by the maintenance dose after 10 days, and subsequently on a monthly basis. Purified aqueous extracts of Vespula venom (ALK-Abelló) were used during the two-day induction phase, followed by depot formulations for the subsequent maintenance phase. (1)*

**Table S2 Baseline characteristics (N = 24)**

| **Variable** | **Value** |
| --- | --- |
| Age (years) | 60 [50.5 – 67] |
| Sex (male/female) | 22 (92%)/ 2 (8%) |
| Occupational exposure risk | 8 (33%) |
| Comorbidities |  |
| – Atopic status | 1 (4%) |
| – Cardiovascular | 11 (46%) |
| – Metabolic | 7 (29%) |
| – Respiratory | 3 (13%) |
| **Index reaction (IR)** |  |
| – Mueller I | 2 (8%) |
| – Mueller II | 6 (25%) |
| – Mueller III | 5 (21%) |
| – Mueller IV | 11 (46%) |
| – Hypotension | 9 (38%) |
| – Multiple stings | 3 (13%) |
| **Insect involved** |  |
| — Vespula sp. | 5 (21%) |
| — Vespa crabro | 19 (79%) |
| **Other HVA** | No |
| **Before Reaction** |  |
| – Previous stings by the same insect | 23 (96%) |
| – Previous systemic reaction | 4 (17%) |
| – Time since IR (months) | 12.8 [1.7 – 59.9] |
| **Allergy diagnostic testing** |  |
| – Total IgE (kU/L) | 105 [24.2 – 404.5] |
| – Specific IgE to Vespula spp. (kU/L) | 4.2 [1.5 – 9.1] |
| – Specific IgE to Ves v 1 (kU/L) | 1.1 [0.1 – 4.8] |
| – Specific IgE to Ves v 5 (kU/L) | 1.9 [0.8 – 9.6] |
| **Skin test * (mcg/mL)** | 0.01 [0.01 – 0.1] |
| – 0.001 mcg/mL | 2 (6.3%) |
| – 0.01 mcg/mL | 10 (40.6%) |
| – 0.1 mcg/mL | 9 (37.5%) |
| – 1 mcg/mL | 2 (6.3%) |
| **Baseline tryptase (μg/L)** | 4.6 [3.6 – 7.0] |
| **REMA score** | -2 [-2 – +1] |

Data are reported as medians [interquartile range] or percentages.

* Median concentration eliciting positive intradermal skin test and percentage of patients reactive at each concentration.

**R e f e r e n c e s**

1. Bilò M.B., Corsi A., Agolini S., Tontini C., Antonicelli L. [Safety of a 2-day ultrarush immunotherapy in vespid allergic patients: Focus on elevated serumtryptase.](https://pubmed.ncbi.nlm.nih.gov/29577980/) Ann Allergy Asthma Immunol. 2018 Jul;121(1):130-132.
